# Neoadjuvant Chemo-Immunotherapy for Early-Stage Non–Small Cell Lung Cancer: A Systematic Review and Meta-Analysis

**DOI:** 10.1001/jamanetworkopen.2024.6837

**Published:** 2024-04-16

**Authors:** Giuseppe Luigi Banna, Mona Ali Hassan, Alessio Signori, Emilio Francesco Giunta, Akash Maniam, Shobana Anpalakhan, Shyamika Acharige, Aruni Ghose, Alfredo Addeo

**Affiliations:** 1 Portsmouth Hospitals University NHS Trust, Portsmouth, United Kingdom; 2 Faculty of Science and Health, School of Pharmacy and Biomedical Sciences, University of Portsmouth, Portsmouth, United Kingdom; 3 Department of Health Sciences, University of Genoa, Genoa, Italy; 4 Department of Medical Oncology, IRCCS Istituto Romagnolo per lo Studio dei Tumori “Dino Amadori,” Meldola, Italy; 5 Department of Oncology, Southampton General Hospital, Southampton, United Kingdom; 6 Department of Medical Oncology, Barts Cancer Centre, St Bartholomew’s Hospital, London, United Kingdom; 7 Oncology Service, Geneva University Hospital, Geneva, Switzerland

## Abstract

**Importance:**

Randomized clinical trials (RCTs) with neoadjuvant immune checkpoint inhibitors (ICIs) plus chemotherapy (ICI-chemotherapy) for patients with early-stage non–small cell lung cancer (NSCLC) have reported consistent associations with event-free survival (EFS) and pathologic complete response (pCR) pending longer follow-up for overall survival data.

**Objective:**

To assess the pooled benefit of ICI-chemotherapy in 2-year EFS and pCR among patients with NSCLC and examine the impact of clinical, pathologic, and treatment-related factors.

**Data Sources:**

Full-text articles and abstracts in English were searched in EMBASE, PubMed, the Cochrane Central Register of Controlled Trials, and the Cochrane Database of Systematic Reviews through November 1, 2023, and in oncology conference proceedings from January 1, 2008, to November 1, 2023.

**Study Selection:**

Phase 2 or 3 RCTs with neoadjuvant ICI-chemotherapy with or without adjuvant ICIs vs neoadjuvant chemotherapy alone with or without placebo or observation in patients with previously untreated NSCLC staged IB to IIIB were included.

**Data Extraction and Synthesis:**

Data extraction of prespecified data elements was performed by 2 reviewers using a structured data abstraction electronic form. A random-effects model was used for meta-analysis. The meta-analysis followed the Preferred Reporting Items for Systematic Reviews and Meta-Analyses guideline.

**Main Outcomes and Measures:**

Two-year EFS and pCR were the outcomes of interest in patients who received neoadjuvant ICI-chemotherapy (experimental arm) or neoadjuvant chemotherapy alone (control arm). Aggregated pooled hazard ratios (HRs) for time-to-event outcomes (2-year EFS) and risk ratios (RRs) for dichotomous outcomes (pCR) with their respective 95% CIs were calculated.

**Results:**

Eight trials with 3387 patients were included, with some concerns of risk of bias as assessed by the Cochrane Collaboration method, mainly related to outcomes measurements. Neoadjuvant ICI-chemotherapy was associated with improved 2-year EFS (HR, 0.57; 95% CI, 0.50-0.66; *P* < .001) and increased pCR rate (RR, 5.58; 95% CI, 4.27-7.29; *P* < .001) in the experimental vs control treatment arms. This association was not significantly modified by the main patient characteristics; tumor- or treatment-related factors, including tumor programmed cell death ligand 1 (PD-L1) status; type of platinum-compound chemotherapy; number of cycles of neoadjuvant ICI-chemotherapy; or addition of adjuvant ICIs. Patients whose tumor cells were negative for PD-L1 were at higher risk of relapse (HR, 0.75; 95% CI, 0.62-0.91) than were those with low (HR, 0.61; 95% CI, 0.37-0.71) or high PD-L1 (HR, 0.40; 95% CI, 0.27-0.58) (*P* = .005).

**Conclusions and Relevance:**

In this systematic review and meta-analysis of neoadjuvant ICI-chemotherapy RCTs in patients with early-stage NSCLC, 3 cycles of neoadjuvant platinum-based ICI-chemotherapy were associated with a meaningful improvement in 2-year EFS and pCR.

## Key Points

**Question:**

Are neoadjuvant immune checkpoint inhibitors (ICIs) plus chemotherapy associated with improved 2-year event-free survival (EFS) and pathologic complete response (pCR) in patients with early-stage non–small cell lung cancer (NSCLC)?

**Findings:**

This systematic review and meta-analysis of 8 randomized clinical trials including 3387 patients found improved 2-year EFS and increased pCR rate associated with neoadjuvant ICI-chemotherapy vs neoadjuvant chemotherapy alone, irrespective of programmed cell death 1 ligand status, platinum-compound chemotherapy, number of neoadjuvant ICI-chemotherapy cycles, or addition of adjuvant ICIs.

**Meaning:**

The findings suggest that neoadjuvant platinum-based ICI-chemotherapy for patients with early-stage NSCLC is associated with improvements in 2-year EFS and pCR.

## Teaser

This systematic review and meta-analysis examines 2-year event-free survival and pathologic complete response among patients with early-stage non–small cell lung cancer receiving neoadjuvant immune checkpoint inhibitors plus chemotherapy.

## Introduction

Of all non–small cell lung cancer (NSCLC) cases, approximately 50% are diagnosed at an early stage, when surgical resection is a viable treatment option. Among patients with early-stage cases, 20% present with stage I or II disease, while 30% present with stage IIIA or IIIB disease.^1^

Neoadjuvant immune checkpoint inhibitors (ICIs) can improve the surgical resectability of tumors and decrease the risk of distant relapse.^2,3,4,5^ Randomized clinical trials (RCTs) have investigated the use of neoadjuvant ICIs in combination with platinum-based chemotherapy (ICI-chemotherapy) in resectable NSCLC, demonstrating a prolongation of event-free survival (EFS) and a significant increase in pathologic complete response (pCR).^6^ The latter is recognized as a potential surrogate for overall survival (OS).^7,8,9^ This led to the approval of a neoadjuvant ICI regimen for resectable NSCLC by the US Food and Drug Administration and European Medicines Agency.^10,11,12^

Some studies indicated greater benefits in EFS and/or pCR in patients with increased programmed cell death ligand 1 (PD-L1) expression in tumor cells.^13,14,15^ This led the European Medicines Agency to restrict the use of nivolumab as neoadjuvant therapy in combination with platinum-based chemotherapy to patients with resectable NSCLC and tumor cell PD-L1 expression of at least 1%.^12^ Other potential factors related to benefit were histologic features, disease stage, and smoking history.^13,14,15,16^ The role of other factors, like the use of cisplatin- or carboplatin-based chemotherapy, is still uncertain.

Additionally, it is particularly unclear whether adjuvant postoperative ICI treatment is necessary for all patients who receive neoadjuvant ICI-chemotherapy. Moreover, there is uncertainty about the required amount of preoperative ICI-chemotherapy.

We conducted a systematic review and meta-analysis of RCTs with neoadjuvant ICI-chemotherapy with or without adjuvant ICIs to assess the pooled benefit from neoadjuvant ICI-chemotherapy in EFS and pCR in patients with early-stage NSCLC and investigate the impact of various clinical, pathologic, and treatment-related factors.

## Methods

The Preferred Reporting Items for Systematic Reviews and Meta-Analyses (PRISMA) statement was used to report this systematic review and meta-analysis.^17^ The meta-analysis protocol was registered in PROSPERO.

### Study Objectives

The current study aimed to assess the extent of benefit from neoadjuvant ICI-chemotherapy with or without adjuvant ICIs compared with neoadjuvant chemotherapy alone with or without placebo or observation in 2-year EFS and pCR by a systematic review and classic meta-analysis. The 2-year EFS and pCR were the outcomes of interest as efficacy end points.

Secondary objectives included the assessment of any differences in 2-year EFS and pCR benefits from neoadjuvant ICI-chemotherapy with or without adjuvant ICIs across the patient subgroups. Additionally, the difference when using adjuvant ICIs after neoadjuvant ICI-chemotherapy and the number of neoadjuvant ICI-chemotherapy cycles were assessed for 2-year EFS and pCR benefit, respectively.

Therefore, an exploratory analysis of 2-year EFS and pCR by patient subgroups (eMethods in Supplement 1) and the associations of use of adjuvant ICI after neoadjuvant ICI-chemotherapy vs placebo or observation with 2-year EFS and of 3 vs 4 neoadjuvant ICI-chemotherapy cycles with pCR was planned. Considering incompleteness or poor data reporting, particularly for subgroup analyses, outcomes analyses were performed if data from at least 2 studies were available. Outcomes of interest included 2-year EFS and pCR as efficacy end points.

### Eligibility Criteria

Included studies were phase 2 or 3 RCTs testing neoadjuvant ICI-chemotherapy with or without adjuvant ICIs vs neoadjuvant chemotherapy alone with or without placebo or observation in patients at least 18 years of age with previously untreated, pathologically confirmed NSCLC staged IB to IIIB (according to the tumor-node-metastasis staging system based on the *Eighth Edition American Joint Committee on Cancer [AJCC] Cancer Staging Manual*) whose disease was deemed resectable and of any PD-L1 status. Experimental treatment arms were considered those including treatment with ICIs directed against programmed cell death 1, PD-L1, or cytotoxic T-lymphocyte–associated protein 4 given in combination with neoadjuvant platinum-based chemotherapy in any formulation (intravenous or oral doses). Comparators or controls were neoadjuvant platinum-based chemotherapy with or without placebo or adjuvant placebo or observation.

### Data Sources and Search Strategies

A comprehensive literature search was performed in EMBASE, PubMed, the Cochrane Central Register of Controlled Trials, and the Cochrane Database of Systematic Reviews for English-language articles published in print or online in peer-reviewed journals through November 1, 2023; proceedings of main international meetings (ie, American Society of Clinical Oncology, European Society for Medical Oncology, International Association for the Study of Lung Cancer World Conference on Lung Cancer, and European Lung Cancer Congress annual meetings) from January 1, 2008, to November 1, 2023, were included. The detailed search terms and procedures are described in eTable 1 in Supplement 1; the data sources searched are in eTable 2 in Supplement 1.

### Study Selection

Full-text articles and abstracts deemed relevant by screening the list of titles were identified and reviewed by ^2^ individual reviewers (M.A.H., E.F.G.). Disagreements were resolved with consensus.

### Data Extraction

Data extraction from each trial and collection of prespecified data elements were performed by 2 reviewers (G.L.B., A.M.) using a structured data abstraction electronic form created by G.L.B. and checked and queried for consistency by A.S. (eMethods in Supplement 1). Disagreements between the 2 reviewers were resolved by referring to a third reviewer (S. Anpalakhan).

### Risk of Bias

The random sequence generation, allocation concealment, blinding, determination of incomplete outcome data, and selection of outcome reporting domains for assessing the risk of bias in the study were performed using the revised Cochrane risk-of-bias RoB 2 tool.^18,19^ Two independent reviewers (M.A.H., A.S.) separately rated trial quality, and differences were resolved by appealing to a third reviewer (A.A.).

### Statistical Analysis

Aggregated 2-year EFS and pCR, pooled hazard ratios (HRs) for time-to-event outcomes (2-year EFS), and risk ratios (RRs) for dichotomous outcomes (pCR) with their respective 95% CIs were calculated in both the experimental and control arms according to treatment type and patient subgroup. When the HR for 2-year EFS was not available, it was estimated from the Kaplan-Meier curves using the approaches described by Tierney et al^20^ based on the number of censored and at-risk patients and the number of events in a fixed time. As an alternative, if only the number of events and median survival times (MSTs) were reported, the MSTs were used to estimate the hazard rate in each arm (hazard rate = ln(2)/MST) and to derive the HR between the 2 arms. The SE of the log HR for the calculation of the 95% CI was derived by the *Z* value, corresponding to the reported *P* value of the log-rank test, from a table of standard normal distributions (SE = ln(HR)/*Z*). Regarding the dichotomous outcome (pCR) and the RR calculation, in the absence of the number of events, a study was excluded from the analysis for this outcome.

A classic meta-analysis was performed to estimate the pooled effect size of the experimental arm vs the control arm for either the time-to-event outcome (pooled HR) or the dichotomous outcome (pooled RR). To proceed with data synthesis, a minimum of 2 studies was required.

The study effect sizes were synthesized using random-effects models with the restricted maximum likelihood method to account for heterogeneity. Heterogeneity was quantified by the Higgins *I*^2^ coefficient and statistically tested by the Cochrane *Q* test.

Prespecified subgroup analyses were performed to assess the potential association of some clinical and biological factors with the end points according to the subgroups indicated in the eMethods in Supplement 1. For pCR outcomes, in the case of 0 events in 1 or more subgroups, an adjustment was made by adding 0.5 to all cells involved in the RR calculation. In the subgroup analyses, the test for difference between the subgroups pooled effect was reported.

Two-sided *P* < .05 was considered significant for the difference between treatments. Results were reported as conventional meta-analysis forest plots using Stata, version 16 (StataCorp LLC).

## Results

### Study Selection and Characteristics

A total of 1027 titles and abstracts were identified by the electronic screening search, of which 8 references reporting 8 RCTs met the eligibility criteria (eFigure 1 and eTable 1 in Supplement 1).^13,14,15,16,21,22,23,24^ The 8 RCTs included 3387 patients. All trials were designed for testing superiority and included patients of any gender who were aged 18 years or older and had known histologic features (ie, nonsquamous or squamous), PD-L1 tumor expression, and an Eastern Cooperative Oncology Group (ECOG) performance status (PS) score of 0 to 1; trials excluded patients with known *EGFR* alterations or *ALK* translocations. The characteristics of the included RCTs and outcomes of interest are outlined in the Table.

**Table.** Main Characteristics of Included Neoadjuvant ICI-Chemotherapy Randomized Clinical Trials and Outcomes of Interest

Source (phase)	Treatment (No. of cycles)		Primary end point	Patients, No.		2-y EFS (95% CI)		*P* value	pCR rate (95% CI)		*P* value	Surgery, exp vs control, %
	Control	Exp		Control	Exp	Control	Exp		Control	Exp		
Forde et al,^13^ 2022; CheckMate 816 (3)	PBC (3), surgery	Nivolumab, 360 mg, plus PBC (3), surgery	EFS and pCR	179	179	45.3 (NR)	63.8 (NR)	<.001	2.2 (0.6-5.6)	24.0 (18.0-31.0)	<.001	83.2 vs 75.4
Heymach et al,^21^ 2023; AEGEAN (3)	Placebo plus PBC (4), surgery, and placebo (12)	Durvalumab, 1500 mg, plus PBC (4), surgery, and durvalumab (12)	EFS by BICR; pCR	374	366	52.4 (45.4-59.0)	63.3 (56.1-69.6)	<.001	4.3 (2.5-6.9)	17.2 (13.5-21.5)	<.001	77.6 vs 76.7
Lei et al,^22^ 2023; TD-FOREKNOW (2)	Platinum based chemotherapy with nab-paclitaxel (3), surgery	Camrelizumab, 200 mg, every 3 wk for 3 cycles plus PBC plus nab-paclitaxel (3), surgery	pCR	45	43	67.6 (48.0-81.2)	76.9 (56.3-88.7)	NA	8.9 (2.5-21.2)	32.6 (19.1-48. 5)	<.001	93.0 vs 93.3
Zhang et al,^15^ 2021; Neotorch (3)	Placebo plus PBC (3), surgery, placebo plus PBC (1), and then placebo (13)	Toripalimab, 240 mg, every 3 wk plus PBC (3), surgery, toripalimab, 240 mg, every 3 wk plus PBC (1), and toripalimab, 240 mg (13)	EFS in stage III by investigators; mPR evaluated by BIPR in stage III	202	202	38.7 (NR)	64.7 (NR)	<.001	1 (0.1-3.5)	28.2 (22.1-35.0)	<.001	82.2 vs 73.3
Provencio et al,^23^ 2023; NADIM II (2)	PBC (3), surgery, and observation	Nivolumab, 360 mg, plus PBC (3), surgery, and nivolumab, 480 mg (6)	pCR	29	57	40.9 (26.2-63.6)	67.2 (55.8-81.0)	NA	7 (1-23)	37 (24-51)	.02	93.0 vs 69.0
Wakelee et al,^14^ 2023; KEYNOTE-671 (3)	Placebo plus PBC (4), surgery, and placebo (13)	Pembrolizumab, 200 mg, plus PBC (4), surgery, and pembrolizumab, 200 mg (13)	EFS and OS	400	397	40.6 (34.8-46.3)	62.4 (56.8-67.5)	<.001	4 (2.3-6.4)	18.1 (14.5-22.3)	<.001	82.1 vs 79.4
Cascone et al,^16^ 2023; CheckMate 77T (3)	Placebo plus PBC (4), surgery, and placebo (12)	Nivolumab, 360 mg, plus PBC (4), surgery, and nivolumab, 480 mg (12)	EFS by BICR	232	229	50.0 (NR)	70.0 (NR)	<.001	4.7 (2.4-8.3)	25.3 (19.8-31.5)	NR	78.0 vs 77.0
Yue et al,^24^ 2023; RATIONALE-315 (3)	Placebo plus PBC (4), surgery, and placebo (8)	Tislelizumab, 200 mg, plus PBC (3), surgery, and tislelizumab, 400 mg (8)	mPR by BIPR and EFS by BICR	227	226	NR	NR	NA	5.7 (NR)	40.7 (NR)	<.001	84.1 vs 76.2

Abbreviations: BICR, blinded independent central review; BIPR, blinded independent central pathology review; EFS, event-free survival; Exp, experimental; ICI, immune checkpoint inhibitor; mPR, major pathologic response; NA, not applicable; NR, not reported; OS, overall survival; PBC, platinum-based chemotherapy; pCR, pathologic complete response.

Five^13,14,15,16,24^ of the 8 RCTs were placebo-controlled in the neoadjuvant treatment; the phase 2 TD-FOREKNOW^22^ and NADIM II^23^ trials were not, while the CheckMate 816^13^ trial was the only open-label phase 3 study. Except for the CheckMate 816^13^ and TD-FOREKNOW^22^ trials, all the other studies^14,15,16,21,23,24^ included adjuvant ICI therapy, which was placebo-controlled in all except the NADIM II^23^ trial. The Neotorch^15^ trial was the only study including adjuvant ICI-chemotherapy followed by ICI.

In 4 RCTs,^13,15,22,23^ 3 cycles of neoadjuvant ICI-chemotherapy were given, while 4 cycles were given in the other 4 studies.^14,16,21,24^ The ICI used was an anti-PD1 agent (namely, camrelizumab, nivolumab, pembrolizumab, tislelizumab, or toripalimab) in all trials^13,14,15,16,21,22,23,24^ except the AEGEAN^21^ trial, in which an anti–PD-L1 agent was investigated (ie, durvalumab).

The primary end point was EFS for 6 of the studies,^13,14,15,16,21,24^ pCR for 4 of the studies,^13,21,22,23^ and major pathologic response for 2 of the studies,^15,24^ while only the KEYNOTE-671^14^ trial included OS and EFS. The EFS was consistently defined as the time from randomization to the first occurrence of disease progression or recurrence or death of any cause by all the studies. However, its definition was not reported either in the relevant abstract or in the oral presentation for the Neotorch^15^ and RATIONALE-315^24^ trials. The EFS assessment was based on blinded, independent, centralized review in the CheckMate 816,^13^ AEGEAN,^21^ and CheckMate 77T^16^ trials. Four of the 8 RCTs included the occurrence of any disease progression precluding surgery in the definition of EFS,^13,14,16,21^ while the time from randomization to the point the tumor was considered unresectable was estimated as part of the EFS by the KEYNOTE-671^14^ and CheckMate 77T^16^ trials only (eTable 3 in Supplement 1).

In all the RCTs,^13,14,15,16,21,22,23,24^ pCR was defined as the absence of residual vital tumor cells in the primary tumor and surgically removed or sampled lymph nodes, and patients who did not undergo surgery were considered nonresponders. A blinded independent central review of pCR was carried out in the KEYNOTE-671^14^ and NADIM II^23^ trials, and in 4 studies (CheckMate 816,^13^ Neotorch,^15^ CheckMate 77T,^16^ and RATIONALE-315^24^), the pCR assessment was based on a blinded independent pathologic review (eTable 3 in Supplement 1).

### Two-Year EFS and pCR by Systematic Review

Seven of the RCTs reported EFS data,^13,14,15,16,21,22,23^ but for the RATIONALE-315 trial,^24^ these data were not available yet. All 7 RCTs with EFS data consistently reported higher 2-year EFS rates with neoadjuvant ICI-chemotherapy (from 62.4% to 76.9%) compared with chemotherapy with or without placebo (from 38.7% to 67.6%), with significant differences in all 5 RCTs performing a statistical comparison^13,14,16,22,23^ (Table). The pCR rate was reported by all 8 RCTs and was consistently and significantly higher with neoadjuvant ICI-chemotherapy (from 17.2% to 40.7%) compared with chemotherapy with or without placebo (from 1.0% to 8.9%) (Table).

Despite the CheckMate 816^13^ and the TD-FOREKNOW^22^ trials not including an adjuvant ICI with or without the chemotherapy phase, the reported 2-year EFS rates in the experimental arms (63.8% and 76.9%, respectively) did not seem to differ numerically from those in the other studies^14,15,16,21,23,24^ (in which it ranged from 62.4% to 70.0%). Additionally, the pCR rates did not appear numerically inferior in the 4 aforementioned RCTs giving 3 cycles of neoadjuvant ICI-chemotherapy^13,15,16,21^ (from 24.0% to 40.7%) compared with those giving 4 cycles^14,22,23,24^ (from 17.2% to 25.3%) (Table).

### Two-Year EFS and pCR by Meta-Analysis

**Figure 1.**
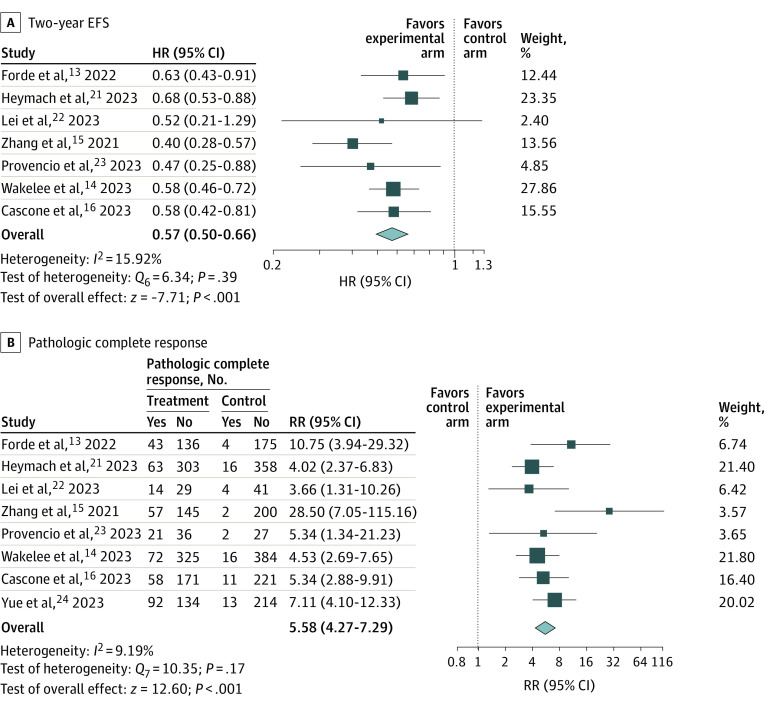
Outcomes by Meta-Analysis Random-effects restricted maximum likelihood model. EFS indicates event-free survival; HR hazard ratio; RR, relative risk.

In the meta-analysis, experimental treatments with neoadjuvant ICI-chemotherapy and adjuvant ICIs with or without chemotherapy were significantly associated with better 2-year EFS (HR, 0.57; 95% CI, 0.50-0.66; *P* < .001) (Figure 1A) compared with control arms with neoadjuvant chemotherapy with or without adjuvant chemotherapy. There was also an association with increased pCR rate (RR, 5.58; 95% CI, 4.27-7.29; *P* < .001) (Figure 1B).

### Subgroup Meta-Analysis by 2-Year EFS and pCR

**Figure 2.**
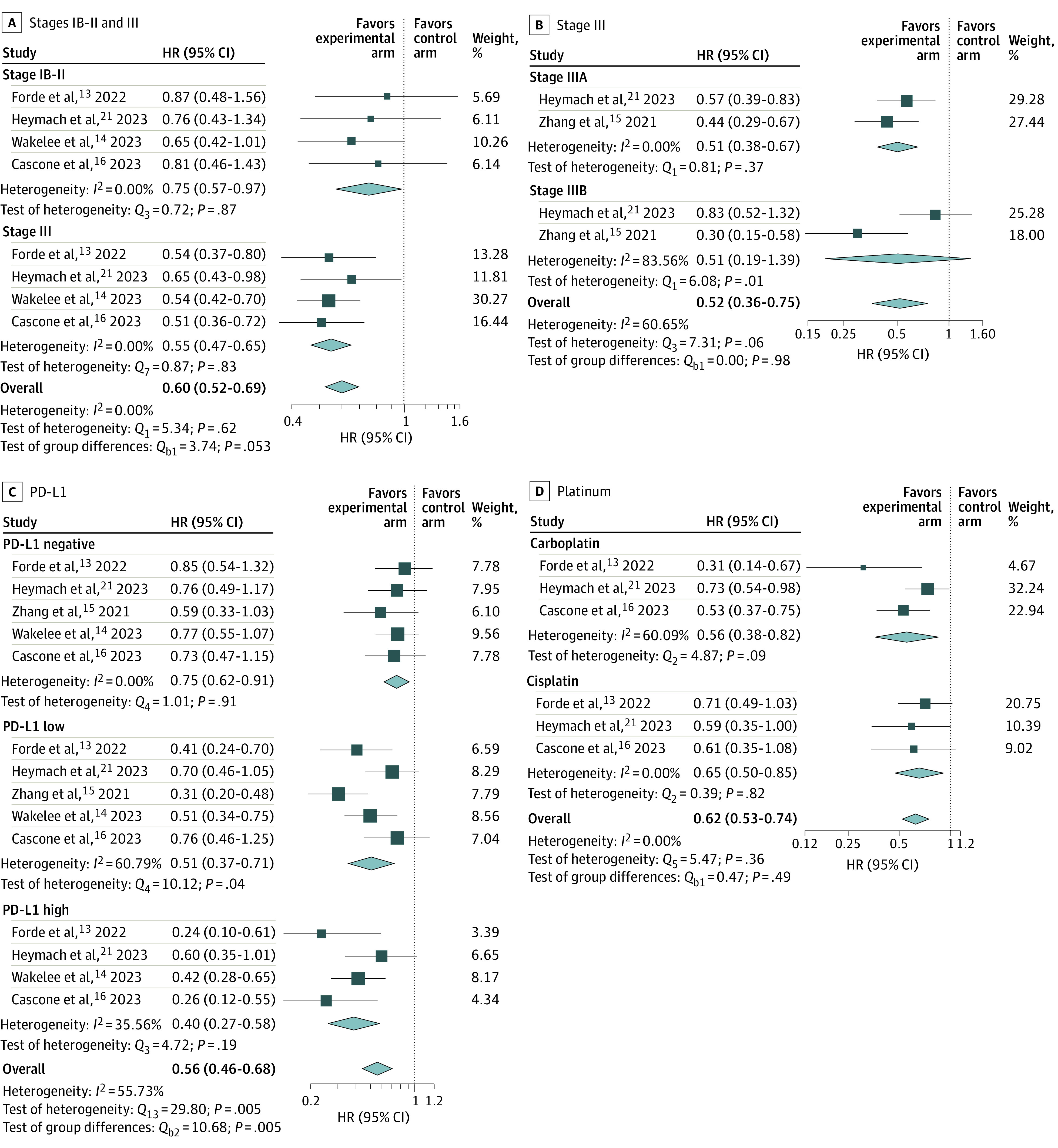
Subgroup Meta-Analysis of 2-Year Event-Free Survival Random-effects restricted maximum likelihood model. HR indicates hazard ratio; PD-L1, programmed cell death ligand 1.

In the subgroup meta-analysis, the association with 2-year EFS observed in the experimental compared with the control arms was irrespective of sex, age, ECOG PS, smoking history, histologic features, tumor stage, and type of platinum-based chemotherapy given (Figure 2 and eFigure 2 in Supplement 1). However, patients with negative PD-L1 tumor status had a greater HR for 2-year EFS (HR, 0.75; 95% CI, 0.62-0.91) compared with those with low (HR, 0.61; 95% CI, 0.37-0.71) or high (HR, 0.40; 95% CI, 0.27-0.58) PD-L1 (*P* = .005 for test of group differences). Furthermore, a lesser HR for the 2-year EFS was observed in patients with stage III disease (HR, 0.55; 95% CI, 0.47-0.65) compared with those with stage IB-II disease (HR, 0.75; 95% CI, 0.57-0.97) (*P* = .053 for test of group differences).

**Figure 3.**
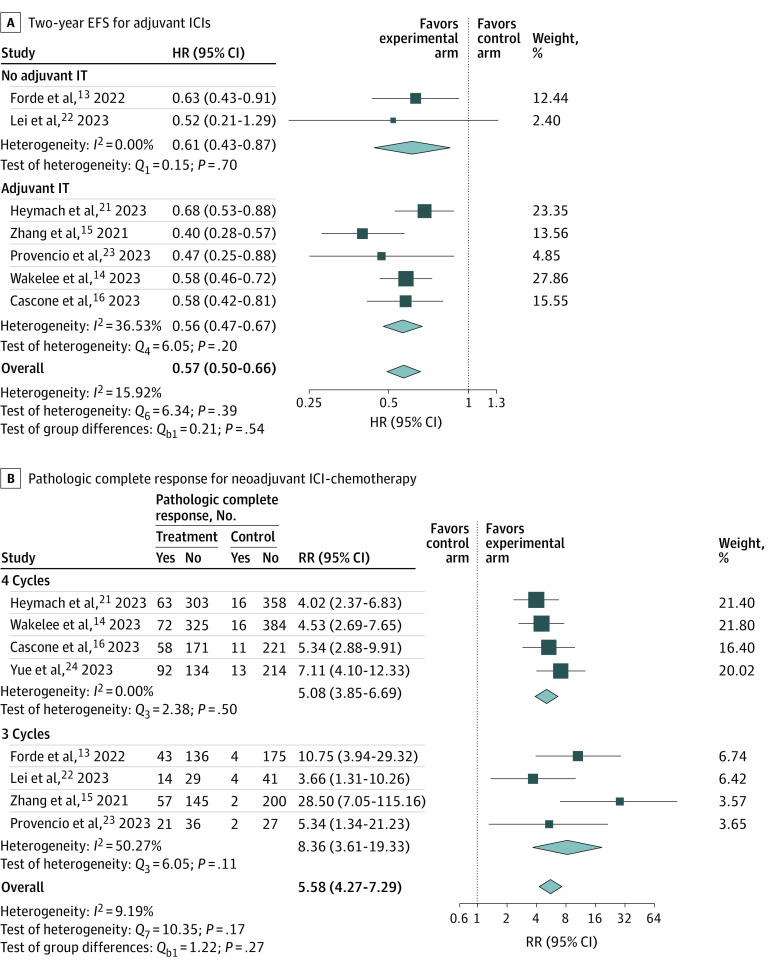
Subgroup Meta-Analysis of 2-Year Event-Free Survival (EFS) and Pathologic Complete Response by Adjuvant Immune Checkpoint Inhibitor (ICI) and Neoadjuvant ICI-Chemotherapy Random-effects restricted maximum likelihood model. HR indicates hazard ratio; IT, immunotherapy; RR, relative risk.

The association with pCR rates observed in the experimental arms compared with the control arms was irrespective of sex, age, ECOG PS, smoking history, histologic features, PD-L1 tumor status, tumor stage, and type of platinum-based chemotherapy given (eFigures 3 and 4 in Supplement 1). No difference in 2-year EFS was observed when using adjuvant ICIs with or without chemotherapy compared with placebo or observation. Additionally, there was no difference in the pCR rate when 3 or 4 cycles of neoadjuvant ICI-chemotherapy were given (Figure 3).

### Risk of Bias

The overall risk of bias raised some concerns, mainly related to the measurement of the outcomes, deviations from intended interventions, and missing outcome data. The CheckMate 816,^13^ NADIM II,^23^ and TD-FOREKNOW^22^ trials were at particularly high risk of bias for deviations from intended interventions and measurement of the outcomes and the TD-FOREKNOW^22^ trial, for missing outcome data (eFigure 5 in Supplement 1).

## Discussion

In less than 3 years, 8 RCTs indicated a significant benefit from combined neoadjuvant chemoimmunotherapy.^13,14,15,16,21,22,23,24^ However, only 1 of these trials, the KEYNOTE-671 trial,^14^ included OS as 1 of the primary end points and recently confirmed its significant prolongation with 4 cycles of neoadjuvant pembrolizumab plus cisplatin-based chemotherapy followed by 9 months of adjuvant pembrolizumab. The other studies^13,15,16,21,22,23,24^ investigated pCR or major pathologic response and/or EFS as primary end points with different therapeutic strategies, such as not including adjuvant ICI therapy, using 3 or 4 neoadjuvant ICI-chemotherapy cycles, or using either carboplatin- or cisplatin-based chemotherapy. Moreover, EFS and particularly pCR are considered reliable surrogates for OS.^7,8,9^

We estimated and quantified a consistent association between neoadjuvant ICI-chemotherapy and 2-year EFS and pCR across the 8 RCTs so far reported.^13,14,15,16,21,22,23,24^ This association was irrespective of patients’ clinical characteristics, tumor features, main prognostic factors, and type of platinum-based chemotherapy for the neoadjuvant chemoimmunotherapy regimen, although the HRs varied in some subgroups. For instance, patients with stage III disease had numerically better HRs and 95% CIs for 2-year EFS than those with stage IB-II disease. Furthermore, we attempted to answer 2 important open questions regarding the use of adjuvant ICI with or without chemotherapy following neoadjuvant ICI-chemotherapy and the number of neoadjuvant ICI-chemotherapy cycles (namely, 3 vs 4). We assumed that any difference for the former should be reflected in the EFS rate and in the pCR rate for the latter, and we could not find any difference for both. This finding suggests that adjuvant ICIs with or without chemotherapy might not be necessary in unselected patients, and 3 cycles of neoadjuvant ICIs with or without chemotherapy may be preferred to 4 cycles. Although only 2 RCTs did not include adjuvant ICIs^13,15^ and we assessed the TD-FOREKNOW^22^ trial as being at high risk of bias (and its results had not been published as of the time of this writing), our findings suggest that additional adjuvant ICIs should be customized according to individual patient circumstances and, in particular, for those at higher risk of relapse. Promising biomarkers for future escalation or deescalation of therapy, such as quantitative and qualitative assessment of circulating tumor DNA, or trials of new combinations with neoadjuvant or perioperative ICIs could be used to avoid unnecessary toxic effects and costs.^25,26^

Unlike other meta-analyses that aimed to compare different perioperative ICI-based therapies,^27,28^ the current analysis did not focus on this comparison. This choice was made to better assess comparable strategies and populations, thus limiting the heterogeneity and providing consistent and clinically helpful evidence from the subgroup analysis. The heterogeneity was low for the 2 primary end points that we studied in the whole population and in the different subgroups, with the only exception being tumor PD-L1 status for 2-year EFS, which suggests caution in the interpretation of the reported association with the 2-year EFS reported in patients with negative PD-L1 tumor status.

Furthermore, despite aiming to focus on 2 reliable efficacy end points, we noticed variability in their assessment across the different RCTs, which must be acknowledged as it was 1 of the main determinants of the concern for the risk of bias derived by the relative qualitative assessment and should inform the design of future clinical trials in the perioperative setting. When surgery is an expected outcome in tumors deemed resectable before the study treatment is given, EFS should specifically also include the time to the point when tumors are considered unresectable alongside disease progression or recurrence that prevents patients from receiving surgery. This was correctly included in the KEYNOTE-671^14^ and CheckMate 77T^16^ trials only (eTable 3 in Supplement 1). Similarly, while pCR was consistently defined as the absence of residual vital tumor cells in the primary tumor and surgically removed or sample lymph nodes, its assessment was not always based on a blinded assessment.

### Limitations

There are limitations to this systematic review and meta-analysis. First, it was based on published RCTs rather than individual patient data. Second, 2-year EFS was used as a benchmark and surrogate end point for OS. However, recent findings from the IFCT-0302 trial^29^ indicated that a majority (66%) of disease relapses in patients with early-stage NSCLC occurred within the first 2 years following surgery. Furthermore, approximately 50% of patients in that trial received either preoperative or postoperative treatments.

## Conclusions

In this systematic review and meta-analysis of neoadjuvant ICI-chemotherapy RCTs in patients with early-stage NSCLC receiving neoadjuvant ICI-chemotherapy, neoadjuvant ICI-chemotherapy was associated with improved 2-year EFS and pCR. This association was not significantly modified by the main patient characteristics or tumor- or treatment-related factors, including high or low tumor PD-L1 status. Three cycles of neoadjuvant ICI-chemotherapy might be preferred to 4, while carboplatin-based regimens seemed equivalent to cisplatin-based ones. There appeared to be no need for additional adjuvant ICIs in the overall population, but this should be customized based on an individual’s biomarker-based risk of relapse.

## Notes

Supplement 1.
eMethods.
**eTable 1.** Search Terms and Results for Each Electronic Database **eTable 2.** Data Sources Searched **eTable 3.** Assessment of EFS and pCR in RCTs of Neoadjuvant ICI-Chemotherapy **eFigure 1.** PRISMA Flow Diagram of Randomized Clinical Trials with Neoadjuvant ICI-Chemotherapy **eFigure 2.** Subgroup Meta-Analysis of 2-Year EFS **eFigure 3.** Subgroup Meta-Analysis of pCR by Sex, Smoking Status, Age, Histology, and Performance Status **eFigure 4.** Subgroup Meta-Analysis of pCR by Tumor Stage, PD-L1 Status, and Platinum-Based Chemotherapy **eFigure 5.** Risk-of-Bias Graph for Review Authors’ Judgements About Each Risk-of-Bias Item Presented as Percentages Across All Included Studies

Supplement 2.
Data Sharing Statement
