# Supplementary material for: Neoadjuvant Chemo-Immunotherapy for Early-Stage Non–Small Cell Lung Cancer: A Systematic Review and Meta-Analysis
Source: JAMA Netw Open. 2024 Apr 16;7(4):e246837. doi: 10.1001/jamanetworkopen.2024.6837 (PMC11022115; doi:10.1001/jamanetworkopen.2024.6837)
Supplement: Supplement 1. — eMethods. eTable 1. Search Terms and Results for Each Electronic Database eTable 2. Data Sources Searched eTable 3. Assessment of EFS and pCR in RCTs of Neoadjuvant ICI-Chemotherapy eFigure 1. PRISMA Flow Diagram of Randomized Clinical Trials with Neoadjuvant ICI-Chemotherapy eFigure 2. Subgroup Meta-Analysis of 2-Year EFS eFigure 3. Subgroup Meta-Analysis of pCR by Sex, Smoking Status, Age, Histology, and Performance Status eFigure 4. Subgroup Meta-Analysis of pCR by Tumor Stage, PD-L1 Status, and Platinum-Based Chemotherapy eFigure 5. Risk-of-Bias Graph for Review Authors’ Judgements About Each Risk-of-Bias Item Presented as Percentages Across All Included Studies [file jamanetwopen-e246837-s001.pdf]

# Supplementary Online Content

Banna GL, Hassan MA, Signori A, et al. Neoadjuvant chemo-immunotherapy for early-stage non–small cell lung cancer: a systematic review and meta-analysis. *JAMA Netw Open*. 2024;7(4):e246837. doi:10.1001/jamanetworkopen.2024.6837

**eMethods**
**eTable 1.** Search Terms and Results for Each Electronic Database
**eTable 2.** Data Sources Searched
**eTable 3.** Assessment of EFS and pCR in RCTs of Neoadjuvant ICI-Chemotherapy
**eFigure 1.** PRISMA Flow Diagram of Randomised Clinical Trials with Neoadjuvant ICI-Chemotherapy
**eFigure 2.** Subgroup Meta-Analysis of 2-Year EFS
**eFigure 3.** Subgroup Meta-Analysis of pCR by Sex, Smoking Status, Age, Histology, and Performance Status
**eFigure 4.** Subgroup Meta-Analysis of pCR by Tumor Stage, PD-L1 Status, and Platinum-Based Chemotherapy
**eFigure 5.** Risk-of-Bias Graph for Review Authors' Judgements About Each Risk-of-Bias Item Presented as Percentages Across All Included Studies

This supplementary material has been provided by the authors to give readers additional information about their work.

© 2024 Banna GL et al. *JAMA Network Open*.

**eMethods**

*Patient subgroups analysed*: sex (male vs female), age (≥65 vs <65), Eastern Cooperative Oncology Group Performance Status (ECOG PS) (0 vs 1), histology (non-squamous vs squamous), PD-L1 tumour expression (negative [0%] vs low [1-49%] vs high [≥50%], disease stage (IB-II vs III, and IIIA vs IIIB), smoking history (current vs vs never smokers), type of platinum-based chemotherapy (cisplatin- vs carboplatin-based) and by the use of adjuvant ICI after neoadjuvant ICI-chemotherapy (for 2yr-EFS only) and 3 vs 4 cycles of neoadjuvant ICI-chemotherapy cycles (for pCR only).

*Data extracted and collected from each trial:* first author, phase of the study, and year of publication/presentation; type of agent, formulation, dose, schedule; number of patients assigned to the experimental and control treatment and by patients' subgroups (i.e., male vs female, age ≥65 vs <65, ECOG PS 0 vs 1); histology (non-squamous vs squamous); PD-L1 tumour expression (negative vs low vs high, as above defined); disease stage (IB-II vs III, and IIIA vs IIIB); smoking status (current vs former vs never); type of platinum-based chemotherapy (cisplatin- vs carboplatin-based); use of adjuvant ICI after neoadjuvant ICI-chemotherapy (vs placebo or observation); number of neoadjuvant ICI-chemotherapy cycles (3 vs 4); study primary endpoint(s) and events needed; study accrual starting and end date; median follow-up and range of experimental and control arms; 2yr-EFS, median and range, number of events, hazard ratio (HR), lower and higher limits of 95% confidence intervals (95% CI), p-value, in the intention to treat population and according to patients' subgroups, in the experimental and control arm; pCR, number of events and assessable patients in experimental and control arm.

© 2024 Banna GL et al. *JAMA Network Open*.

*eTable 1 - Search terms and results for each electronic database (on date 1st Nov 2023)*

| Step | Search | EMBASE (n. of results) | PubMed (n. of results) | Cochrane Library (n. of results) |
|---|---|---|---|---|
| 1 | (non small cell* OR nonsmall cell* OR non-small cell* OR large cell* OR squamous cell* OR squamous small cell* OR nonsquamous cell* OR non-squamous cell* OR nonsquamous small cell* OR non-squamous small cell* OR epidermoid) AND (cancer* OR carcin* OR tumor* OR tumour* OR neoplas* OR oncol* OR malignan*) | | | |
| 2 | (lung* OR pulmonary OR bronchial OR bronchus) | | | |
| 3 | 1 AND 2 | | | |
| 4 | (lung* OR pulmonary OR bronchial) AND (adenocarcin* OR adenocancer*) | | | |
| 5 | NSCLC* | | | |
| 6 | 3 OR 4 OR 5 | 330316 | 182427 | 19176 |
| 7 | (randomised OR randomized OR controlled) AND (clinical trial OR trial OR study) | | | |
| 8 | (neoadjuvant OR neo-adjuvant OR perioperative) | | | |
| 9 | (chemoimmunotherapy OR chemo-immunotherapy OR (chemotherapy AND immunotherapy) OR immunotherapy OR pembrolizumab OR nivolumab OR atezolizumab OR durvalumab OR camrelizumab OR toripalimab OR tislelizumab) | | | |
| 10 | 7 AND 8 AND 9 | 4368 | 618 | 1196 |
| 11 | 6 AND 10 | | | |
| 12 | Animals NOT Humans | | | |
| 13 | (news OR comment OR letter OR editorial OR case report*) | | | |
| 14 | 11 NOT (12 OR 13) | 711 | 83 | 159 |
| 15 | Total number of results (PubMED + EMBASE + Cochrane) | 953 | | |
| 16 | Duplicates identified and deleted | 197 | | |
| 17 | Non duplicated results | 756 | | |

© 2024 Banna GL et al. *JAMA Network Open*.

*eTable 2 - Data sources searched*

| **Database/Information source** | **Interface/Address** | **Search Date** |
|---|---|---|
| **Electronic Databases** | | |
| Cochrane Central Register of Controlled Trials (CENTRAL) and Cochrane Database of Systematic Reviews (CDSR) | Cochrane Library/Wiley: https://www.cochranelibrary.co m | 1st Nov 2023 |
| PubMed | https://www.ncbi.nlm.nih.gov/pubmed | 1st Nov 2023 |
| EMBASE (including MEDLINE) | https://www.embase.com | 1st Nov 2023 |
| **Conference Proceedings / Abstracts from International Meetings** | | |
| American Association for Cancer Research (AACR) | Searched via the EMBASE database (if the conference was indexed in EMBASE) or via the conference website or journal webpages (if free to access) | 1st Nov 2023 |
| American Society of Clinical Oncology (ASCO) | Searched via the EMBASE database (if the conference was indexed in EMBASE) or via the conference website or journal webpages(if free to access) | 1st Nov 2023 |
| European Lung Cancer Conference (ELCC) | Searched via the EMBASE database (if the conference was indexed in EMBASE) or via the conference website or journal webpages (if free to access) | 1st Nov 2023 |
| European Society for Medical Oncology (ESMO) | Searched via the EMBASE database (if the conference was indexed in EMBASE) or via the conference website or journal webpages(if free to access) | 1st Nov 2023 |
| World Conference on Lung Cancer(WCLC) of the International Association for the Study of Lung Cancer (IASLC) | Searched via the EMBASE database (if the conference was indexed in EMBASE) or via the conference website or journal webpages(if free to access) | 1st Nov 2023 |

© 2024 Banna GL et al. *JAMA Network Open*.

*eTable 3 - Assessment of EFS and pCR in RCTs of neoadjuvant ICI-chemotherapy*

| **eTable3. Assessment of EFS and pCR in RCTs of neoadjuvant ICI-chemotherapy** | | | | | |
|---|---|---|---|---|---|
| **EFS** | | | | | |
| **Study reference, year Study name** | **BICR** | **TFR to first PD/DR** | **TFR to death (any cause)** | **TFR to PD precluding surgery** | **TFR to unresectable tumour** |
| Forde PM et al[13], 2022 CheckMate 816 | Y | Y | Y | Y | NS |
| Heymach JV et al[14], 2023 AEGEAN | Y | Y | Y | Y | NS |
| Lei J et al[15], 2023 TD-FOREKNOW | N | Y | Y | NS | NS |
| Zhang Y et al[16], 2023 Neotorch | N | NR | NR | NR | NR |
| Provencio M et al[20], 2023 Nadim II | N | Y | Y | NS | NS |
| Wakelee H et al[21], 2023 Keynote 671 | N | Y[a] | Y | Y | Y |
| Cascone T et al[22], 2023 CheckMate 77T | Y | Y | Y | Y | Y |
| Yue D et al[23], 2023 RATIONALE 315 | N | NR | NR | NR | NR |
| | | | | | |
| **pCR** | | | | | |
| **Study reference, year Study name** | **BICR** | **BIPR** | **0% residual VTC primary tumour** | **0% residual VTC sampled/ surgically removed lymph nodes** | **Patients who did not undergo surgery as non-responders** |
| Forde PM et al[13], 2022 CheckMate 816 | N | Y | Y | Y | Y |
| Heymach JV et al[14], 2023 AEGEAN | N[b] | N | Y | Y | Y |
| Lei J et al[15], 2023 TD-FOREKNOW | N | N | Y | Y | Y |
| Zhang Y et al[16], 2023 Neotorch | N | Y | NR | NR | Y |
| Provencio M et al[20], 2023 Nadim II | Y | N | Y | Y | Y |
| Wakelee H et al[21], 2023 Keynote 671 | Y | Y | Y | Y | Y |
| Cascone T et al[22], 2023 CheckMate 77T | N | Y | Y | Y | Y |
| Yue D et al[23], 2023 RATIONALE 315 | N | Y | Y | Y | Y |
| | | | | | |

Abbreviations: BICR, Blinded Independent Central Review BIPR, Blinded Independent Pathological Review; CR, central review; DR, disease recurrence; EFS, event-free survival; Exp, experimental arm; N, no; NR, not reported; NS, not specified; pCR, pathological complete response; PD, progressive disease; TFR, time from randomization; VTC, viable tumour cells; Y, yes.
according to
[a]According to the Response Evaluation Criteria in Solid Tumors version 1.1
[b]Centralised review only

© 2024 Banna GL et al. *JAMA Network Open*.

*eFigure 1. PRISMA Flow Diagram of Randomised Clinical Trials with Neo-Adjuvant ICI-Chemotherapy*

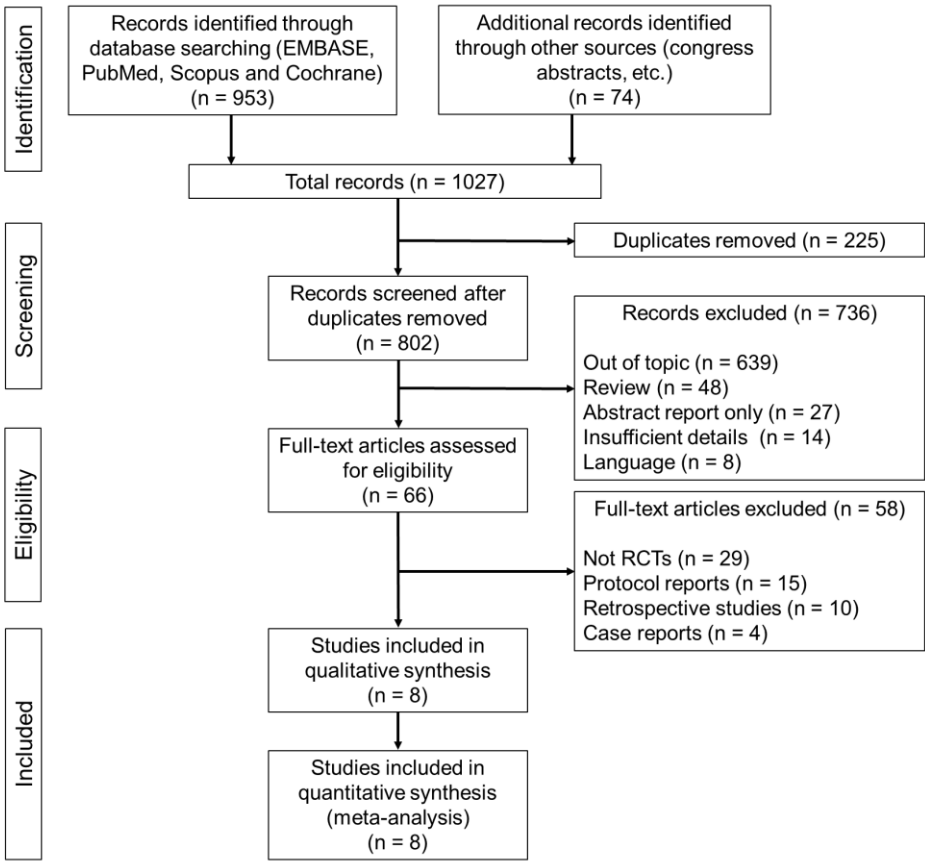


Abbreviations: ICI, immune checkpoint inhibitors; NSCLC, non-small cell lung cancer; PRISMA, Preferred Reporting Items for Systematic Reviews and Meta-Analyses.

© 2024 Banna GL et al. *JAMA Network Open*.

*eFigure 2. Subgroup meta-analysis of 2-yr EFS*

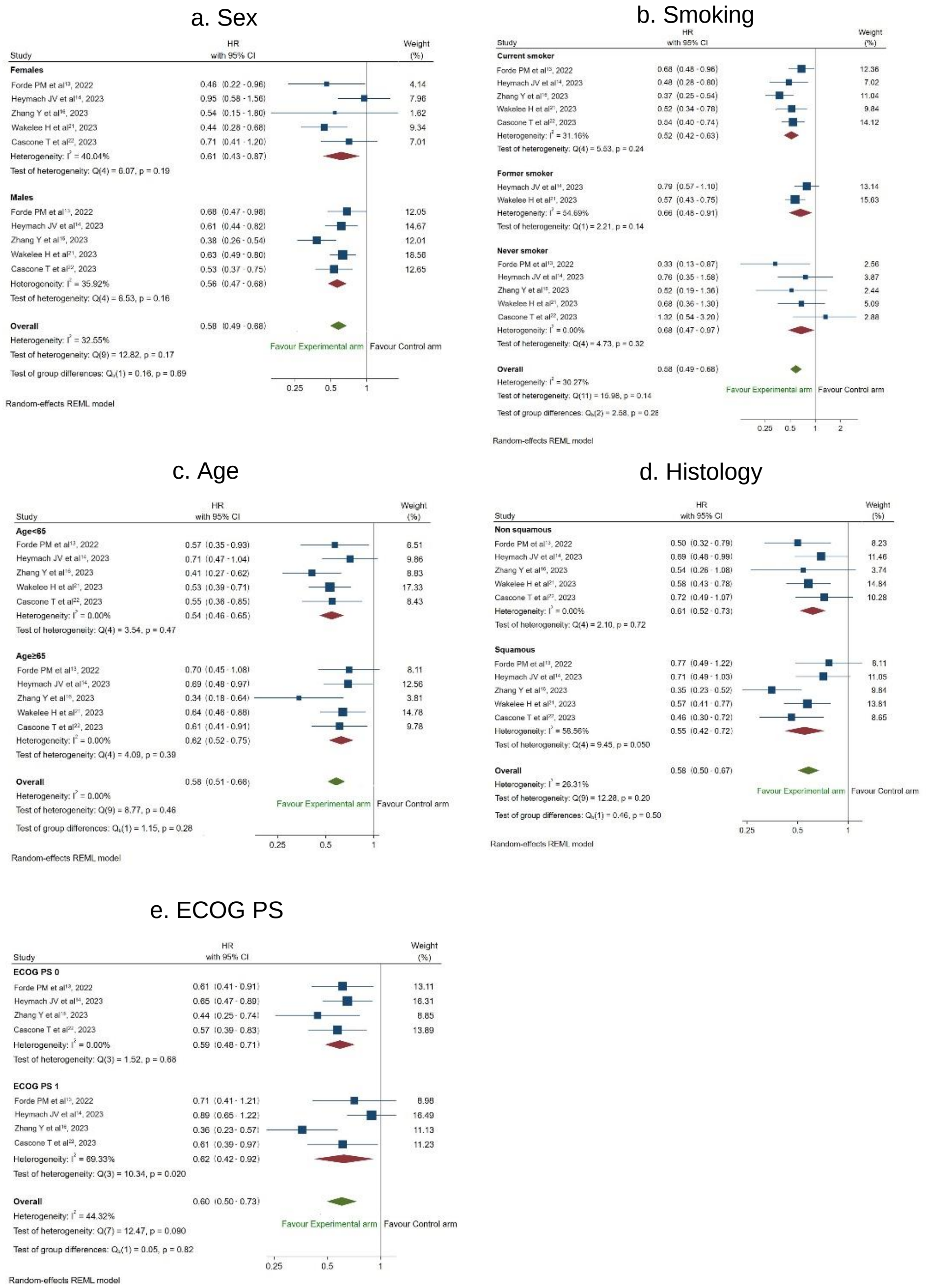


© 2024 Banna GL et al. *JAMA Network Open*.

*eFigure 3. Subgroup meta-analysis of pCR by sex, smoking status, age, histology, and performance status*

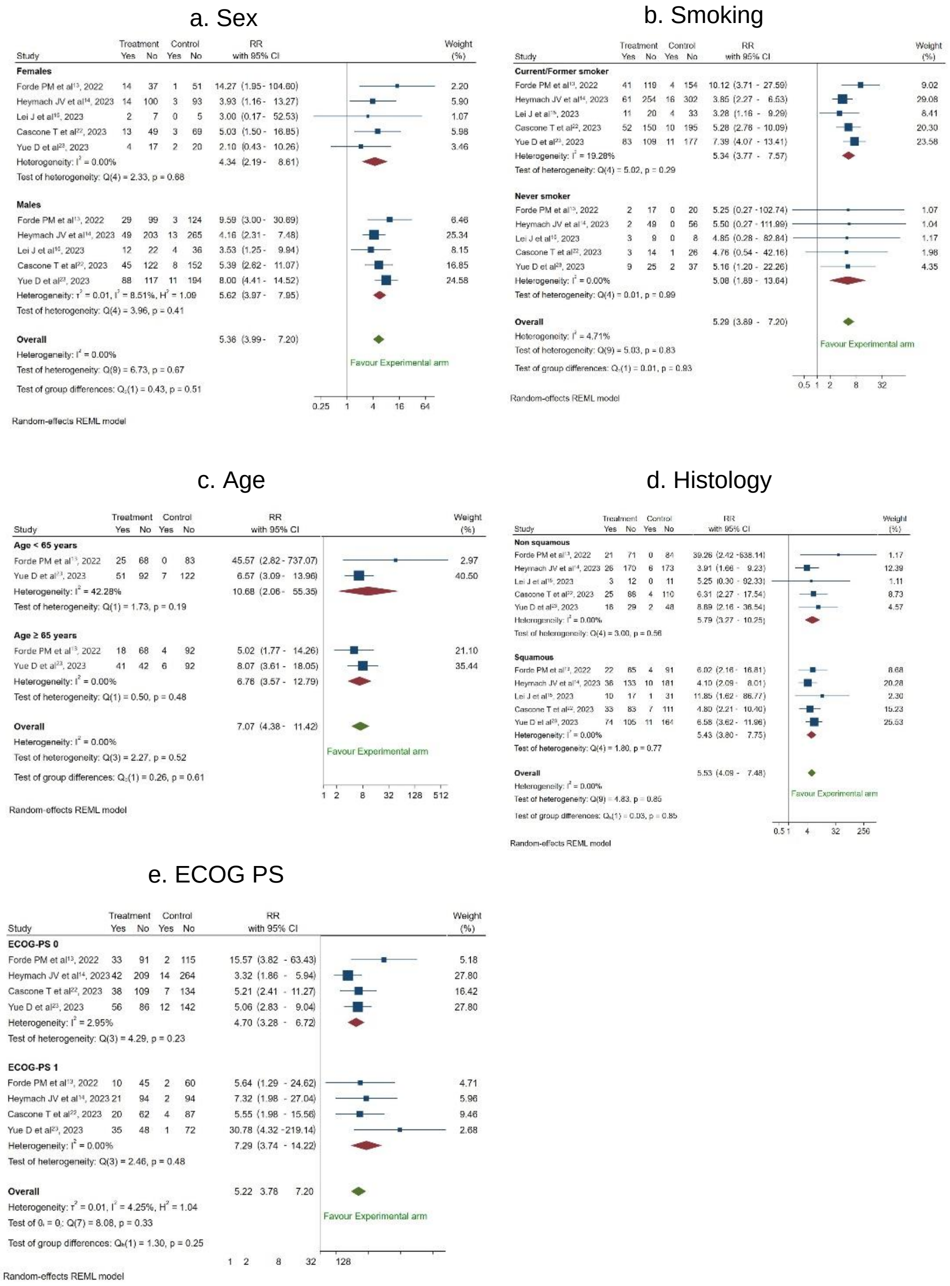


© 2024 Banna GL et al. *JAMA Network Open*.

*eFigure 4. Subgroup meta-analysis of pCR by stage, PD-L1 status, and platinum-based chemotherapy*

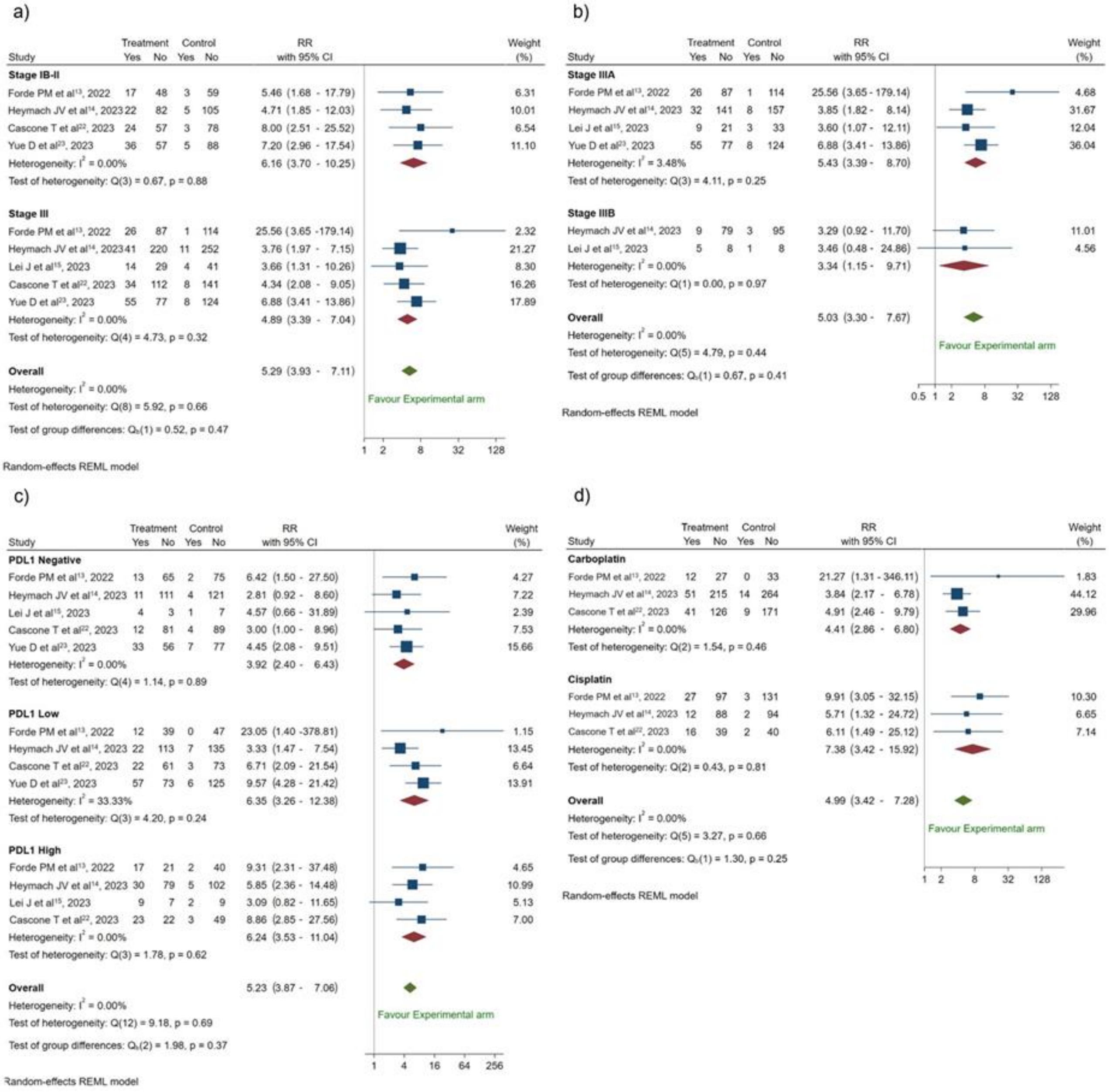


© 2024 Banna GL et al. *JAMA Network Open*.

*eFigure 5. Risk of bias graph: review authors' judgements about each risk of bias item presented as percentages across all included studies*

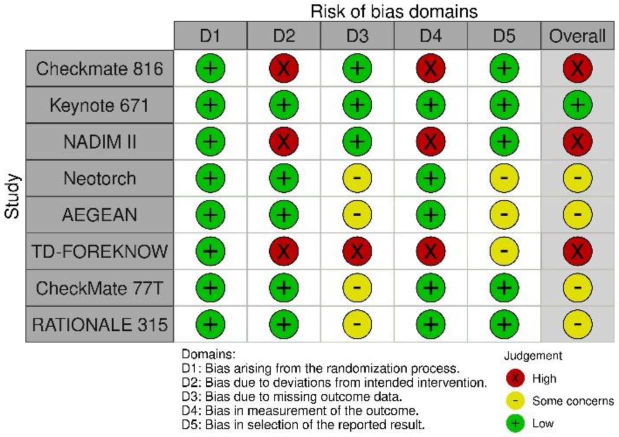


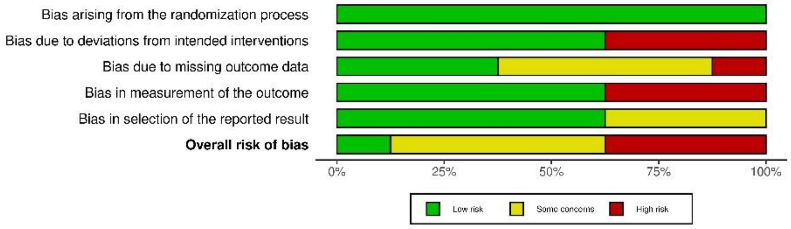


© 2024 Banna GL et al. *JAMA Network Open*.
