# Supplementary material for: Neoadjuvant Chemo-Immunotherapy for Early-Stage Non–Small Cell Lung Cancer: A Systematic Review and Meta-Analysis
Source: JAMA Netw Open. 2024 Apr 16;7(4):e246837. doi: 10.1001/jamanetworkopen.2024.6837 (PMC11022115; doi:10.1001/jamanetworkopen.2024.6837)
Supplement: Supplement 2. — Data Sharing Statement [file jamanetwopen-e246837-s002.pdf]

Data Sharing Statement

Banna. Neoadjuvant Chemo-Immunotherapy for Early-Stage Non–Small Cell Lung Cancer. *JAMA Netw Open*. Published April 16, 2024. doi:10.1001/jamanetworkopen.2024.6837

Data
Data available: No
